# Supplementary material for: The Outcome of Chemotherapy for Metastatic Extramammary Paget’s Disease
Source: J Clin Med. 2021 Feb 12;10(4):739. doi: 10.3390/jcm10040739 (PMC7918098; doi:10.3390/jcm10040739)
Supplement: Supplementary file 1 [file jcm-10-00739-s001.pdf]

Supplementary Table1. Multivariate Cox proportional hazard analyses of overall survival among 21 patients with distant metastasis

Variable	Univariate analysis			Multivariate analysis		
	HR	95% CI	*p*	HR	95% CI	*p*
Sex, male	0.29	0.078–1.05	0.055	0.15	0.014–1.64	0.12
Age (years)†	1.01	0.95–1.07	0.79	0.99	0.93–1.06	0.76
Perianal lesion	0.50	0.12–2.01	0.33	1.78	0.15–21.09	0.65
Chemotherapy, conducted	0.77	0.26–2.25	0.63	1.71	0.30–9.54	0.54
Radiation therapy for metastatic sites, conducted	0.49	0.14–1.75	0.27	0.41	0.11–1.53	0.19

Significant values are presented in boldface.

†Continuous variable.

HR, hazard ratio; CI, confidence interval; LN, lymph node; CLND, completion lymph node dissection

Supplementary Table-2. Treatment-related adverse events

	DTX, n = 12		PTX, n = 2		Low-dose FP, n = 3		S-1, n = 1		Trastuzumab, n = 1	
	Grade 3	Grade 4	Grade 3	Grade 4	Grade 3	Grade 4	Grade 3	Grad 4	Grade 3	Grade 4
Myelosuppression	5	3	1	0	1	1	0	0	0	0
Leukopenia	7	0	1	0	1	1	0	0	0	0
Neutropenia	4	4	1	0	1	1	0	0	0	0
Anemia	1	0	0	0	1	0	0	0	0	0
Thrombocytopenia	0	0	0	0	1	1	0	0	0	0
Febrile neutropenia	2	0	0	0	0	1	0	0	0	0
Renal dysfunction	0	0	0	0	1	0	0	0	0	0
Others	0	0	0	0	0	0	0	0	0	0

DTX, docetaxel; PTX, paclitaxel; FP, 5-fluorouracil + cisplatin
